# Medical students’ career choices, preference for placement, and attitudes towards the role of medical instruction in Ethiopia

**DOI:** 10.1186/s12909-017-0934-z

**Published:** 2017-05-30

**Authors:** Tsion Assefa, Damen Haile Mariam, Wubegzier Mekonnen, Miliard Derbew

**Affiliations:** 1 School of Public Health, Addis Ababa University, PO Box 9086, Addis Ababa, Ethiopia; 2 School of Medicine, Addis Ababa University, PO Box 9086, Addis Ababa, Ethiopia

**Keywords:** Career choice, Ethiopia, Medical students, Medical instruction/education/mentoring, Specialty choice, Practice location, Rural/remote practice

## Abstract

**Background:**

In Ethiopia, the health care delivery and the system of medical education have been expanding rapidly. However, in spite of the expansion, no studies have been carried out among medical students to identify their career choices and attitudes towards the medical instruction. Therefore, this study aimed to fill the gap in evidence in these specific areas.

**Methods:**

Pretested questionnaire was self-administered among fifth and sixth year medical students in six government owned medical schools in Ethiopia. A total of 959 students were involved in the study with a response rate of 82.2%. Career choices, intention where to work just after graduation, and attitudes towards medical instruction were descriptively presented. Binary logistic regression model was fitted to identify factors associated with the intention of medical students to work in rural and remote areas.

**Results:**

Majority, (70.1%) of the medical students wanted to practice in clinical care settings. However, only a small proportion of them showed interest to work in rural and remote areas (21% in zonal and 8.7% in district/small towns). For most, internal medicine was the first specialty of choice followed by surgery. However, students showed little interest in obstetrics and gynecology, as well as in pediatrics and child health as their first specialty of choice.

Medical students’ attitudes towards their school in preparing them to work in rural and remote areas, to pursue their career within the country and to specialize in medical disciplines in which there are shortages in the country were low. The binary logistic regression model revealed that a significantly increased odds of preference to work in rural and remote areas was observed among males, those who were born in rural areas, the medical students of Addis Ababa University and those who had the desire to serve within the country.

**Conclusion:**

This study showed that Ethiopian medical schools are training medical workforce with preferences not to work in rural and remote places, and not to specialize in disciplines where there are shortages in the country. Thus, attention should be given to influence medical students’ attitude to work in rural and remote locations and to specialize in diverse clinical specialties.

**Electronic supplementary material:**

The online version of this article (doi:10.1186/s12909-017-0934-z) contains supplementary material, which is available to authorized users.

## Background

Health workforce education and training is a complex investment, which align education, finance, labor market and policies [1, 2]. In this regard, however, many low-income countries including Ethiopia face significant challenges to train medical doctors [3–5]. Most medical schools have limited residency programs to satisfy the postgraduate career preference, limited role on current and future possibilities (enhancing participation for collective and individual learning) including in providing career and social orientations to medical students [3, 6, 7]. On the other hand, medical students have misunderstandings about the wider picture of medical profession and their personal learning plans [8, 9].

For several years, expansion and development in medical education in Ethiopia was very slow given the fast growing population along with increasing health care demands. However, addressing community health needs were the prime intention of the medical education program since the 1960s, when the country’s first medical faculty was opened with a slogan of ***“the clinical training and internship must be related to the needs of Ethiopia”*** [10]. To date, however, the medical doctors are not available in the communities of rural and remote inhabitants [11–14]. Furthermore, the needs of the medical graduates do not seem to have been addressed, given that many of them have left the country and significant proportion of medical students also intend to leave [15, 16].

**Fig. 1:**
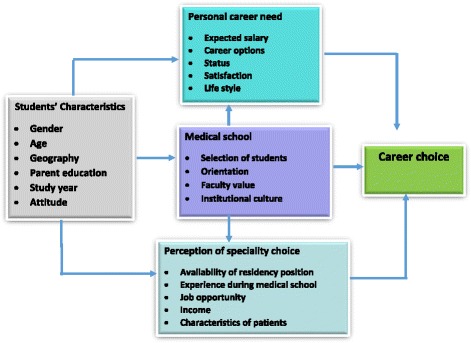
Factors that affect medical students’ career choices (Source: Adopted from Bland CJ, Meurer LN, Maldonado G. Acad. Med. 70:7, 1995)

In professional life, career preferences and choices depend upon different interlinked factors [8, 17, 18]. These include the characteristics of [1] medical schools such as curriculum, orientation to students on career and socialization, recruitment of medical students, faculty values and institutional culture; (2) the characteristics of students such as age, gender, geography and study year; (3) the student values including marital status, academic performance, and attitudes; (4) needs to satisfy for instance expected salary, career options, intellectual satisfaction and workload; (5) perception on specialty characteristics such as availability of positions, and experience during the medical schools; and (6) perception of opportunities such as emigration and working abroad. Figure 1, illustrates the theoretical framework and the interaction among the interlinked factors with career choice [8, 18].

Strengthening medical education is the best strategy to address shortages and distribution of physicians for health care service provision in rural and remote areas. Thus, evidences suggest that the need for putting in place appropriate education and training policies in high-income and low-income countries. For high-income countries not to rely on emigrant physicians [19]; whereas for low-income countries there is a need to retain medical doctors along with strengthening the system of medical education for delivering health care to the rural population and underserved areas [2, 20]. Retention issues are also important in low-income countries since many of their medical graduates want to migrate and pursue their careers abroad [21]. The situation in Ethiopia is not different in this regard despite the limitations of evidences [15].

Understanding medical students’ career preference and intentions where to practice just after graduation is crucial for designing suitable medical school curricula and responding for the requirements of the medical workforce at different stages of their professional life. Therefore, this study aimed to examine medical students’ career choices and attitudes towards medical instruction in preparing the medical students to work in rural and remote locations of Ethiopia.

## Methods

### Setting

Ethiopia has nine regional states and two city administrative councils. The regional states are subdivided into zonal, district (woreda) and kebele (the lowest) structural administrative units. The country has a population of 92 million, of which about 80% reside in rural areas [11].

In addition, the health service delivery is organized into three-tier system. The first level/district health system, comprises of primary hospitals, health centers and health posts, while the second level includes general hospitals (also called zonal hospitals), and the third tier consists of teaching/specialized hospitals. Medical doctors in Ethiopia are deployed at all levels of hospitals, with the exception of primary hospitals for specialists [22].

In Ethiopia, students usually join to the medical schools directly after completion of high school without previous tertiary or college level training. However, in more recent years graduates of other health science disciplines or related fields have been join to the medical schools using the so called “innovative medicine” program. The total length of undergraduate medical education in the country is 6 years.

In the country, there are about 33 universities/colleges that have undergraduate medical education program, of which five are owned by the private sector [22]. Among the medical schools, three have longer training experience: the oldest Addis Ababa University (AAU) was established (in 1964), Gondar (in 1978), and Jimma (in 1983). Mekelle and Hawassa Universities have relatively intermediate experience in medical education (both opened in 2003). Haromaya, Bahir Dar, Arsi, Dire Dawa Universities, and St. Paul’s Millennium Medical College are relatively new in medical education training. All the remaining public medical schools did not have graduating class medical students at the time of the study.

### Population

This cross-sectional survey involved undergraduate medical students who pursue their medical education in government owned higher learning institutions of Ethiopia, and was carried out between February and May 2015. In the survey, fifth year (clinical-II) and final year (internship) medical students were involved (assuming that they have thought over their future career plan than the junior ones) from six randomly selected medical schools/colleges out of the ten medical schools with eligible upper class medical students (interns and clinical-II) at the time of the study. These were Addis Ababa, Haromaya, Mekelle, Hawassa, and Dire Dawa Universities, and St. Paul’s Millennium Medical College.

### Sample size calculation

The sample size was calculated by using a single population proportion formula considering the following assumptions: proportion of medical students’ intention to work abroad (*p* = 53%) [15], and 95% confidence interval at Z α/2 = 1.96 (level of significance). However, in our case given the medical schools’ geographic variations, difference in students’ characteristics/selection and the scope of the study to get adequate sample size for modeling and statistical analysis we used a margin of error of 3% and non-response rate of 10%. Nevertheless, about 959 completed questionnaires were returned out of 1165 questionnaires which were given for medical students that made the response rate 82.2%.

### Data collection

Pretested structured questionnaire was used to collect the data. The questionnaire was prepared by reviewing relevant literatures [15, 23, 24] to gather data on (demographic characteristics, career choices and attitudes towards medical instruction). Before the actual use, senior experts in medical education and research examined the content, language and sequence including suitability to the context of the study, in addition to the pretest which was done among the medical students of Gondar (which excluded from the actual study). Finally, the revised version of the tool was self-administered by the medical students. The data collection process was facilitated by the respective medical school deans and assisted by class/group representatives.

### Measurements

The main dependent variable of the survey was medical students’ intention of placement just after graduation, and the predictor variables were socio-demographic, personal and academic related characteristics. Brief description of some variables is given as follows:

- **Career plan/preference:** was assessed using single item question with list of choices; medical students were asked to indicate their first three career choices in which they were interested in to specialize (such as internal medicine, surgery, gynecology and obstetrics, radiology and so on)
- **Practice location:** refers to the intended place of practice by the medical students just after graduation. Here, practice location is categorized into two: 0 = rural and remote (zonal and district hospitals, and underserved areas) and 1 = urban (big cities where specialized hospitals are found).
- **Medical instruction:** in the survey instrument short description was given to the medical students about the medical instruction (see the appendix). Accordingly, the medical students’ attitudes towards medical instruction was measured using six multi-item questions. Each item has five category of responses (strongly disagree, disagree, neutral, agree and strongly agree).
- Cronbach’s alpha was calculated to evaluate the reliability of the instrument for questions regarding attitudes towards medical instruction. Of six multi-item questions, five of them demonstrated strong internal consistency. For instance, the Cronbach’s alpha in relation to *guidance in the field of medicine* was (0.88); *professional development* (0.85); *research undertaking and ethics* (0.85); *the teaching-learning process* (0.87); and in relation to *orientation towards in country practice* the Cronbach’s alpha was (0.85). However, the scale on the teaching-learning environment was excluded because of low Cronbach’s alpha value, 0.50 (Table 2, Additional file 1).

### Data analysis

The data were entered into EpiData version 3.1 and analyzed with SPSS version 16.0 statistical software. Demographic characteristics of medical students, career preferences, attitudes towards medical instruction were descriptively presented. However, binary logistic regression model was run to identify the predictor variables of medical student’s intention to work in rural and remote areas. Initially, univariate logistic analysis was used to investigate the association between demographic, and medical education related variables with the dependent variable, students’ intention to work in rural and remote areas. And finally, binary logistic regression model was fitted to identify variables that predict medical students’ intention to work in rural and remote areas.

In fitting the model, the dependent variable is coded (rural and remote =0 and urban =1), including other demographic, and medical schools related variables. For instance, gender is coded (male = 1 and female = 2); place of birth: urban (regional town, and zonal town) and rural (born in district and rural village); year of study: (fifth year/C-II =1 and final year/interns =2); medical school: (AAU = 1 and others =2). In all cases, *p* < 0.05 and 95% confidence interval was used to check statistical significance of associations. Finally, Hosmer and Lemeshow test was used to check model adequacy (*p* = 0.583).

### Ethical considerations

The study received permission from the Institutional Review Board (IRB) of the College of Health Sciences, Addis Ababa University (Protocol No.043/14/Sph). The approval from AAU was also accepted for conducting the study in Haromaya, Hawassa, and M St. Paul’s Millennium Medical College. In addition, ethical review boards of University of Gondar (Protocol No. R/C/S/V/P 346/2015) and Mekelle University (Protocol No. ERC 0546/2015) have reviewed the proposal and gave additional ethical permissions to conduct the study in their respective universities. Informed consent was obtained from each study participant. No personal identifier was written during data collection and summary measures were used to interpret findings.

## Results

The mean age of the study participants was 24 + 2.7 years and the higher proportion (56.9%) were below 25 years old. Nearly three fourth of the students, (72.2%) were males, a higher proportion (60.7%) were clinical-II (fifth year) students and the remaining were final year (interns). Also the highest proportion (46.4%) were born in big towns followed by those born in districts (17.3%), rural villages (17.4%), and zonal towns (13.6%).

Table 1, shows medical students’ intended place of practice just after they graduate. The highest proportion (70.1%) of the medical students wanted to practice in clinical care settings. However, only a very small proportion of medical students were intended to work in rural and remote areas (10.8% in zonal hospitals and 5.5% in district/primary hospitals), while the majority (44.8%) of them were interested to work in teaching/specialized hospitals and in big cities of Ethiopia.

**Table 1:** Medical students’ intended places of practice just after graduation, 2015

Variable	Characteristics	Frequency	Percent
Career choice	Patient/Clinical care	672	70.1
	Academic/researcher	162	16.9
	Management	76	7.9
	Others	24	2.5
Setting	Academics/teaching hospitals	430	44.8
	Private -clinical	165	17.2
	NGO-non-clinical	152	15.8
	Zonal hospital	104	10.8
	District hospital	53	5.5
	Management- government	17	1.8
	Others	5	0.5
Place	Big cities	387	40.4
	Zonal towns	201	21.0
	Underserved	113	11.8
	Anywhere	102	10.6
	District capitals	83	8.7
	Do not want to practice	38	4.0
	Other	5	0.5

**Fig. 2:**
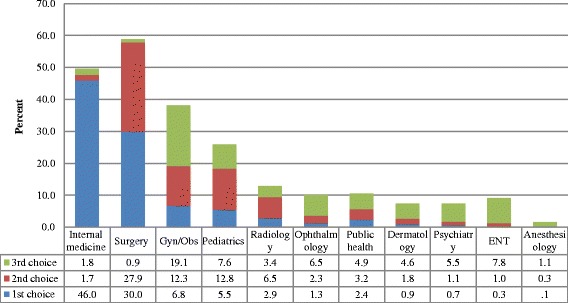
The first three priority of choices to specialize by the medical students in Ethiopia, 2015

**Fig. 3:**
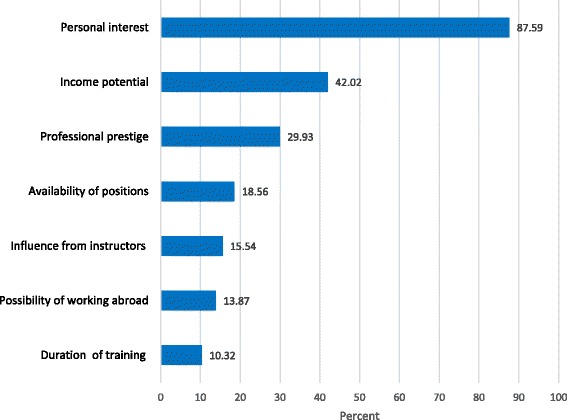
Main reasons for specialty choice among the medical students of Ethiopia, 2015

Figure 2, illustrates the first three selected specialties of choices for postgraduate training. Internal medicine was the first specialty of choice for (46%) of the medical students and surgery was the first for about one third, (30.0%) of the medical students. However, obstetrics and gynecology, and pediatrics and child health were the first choice only for (6.8%) and (5.5%) of the medical students, respectively. The main reasons for specialty preference were personal interest (87.6%), income potential (42%), professional prestige (29.9%), availability of positions (18.6%), and influence from instructors (15.5%) (Fig. 3).

Table 2, shows medical students’ attitudes and opinions towards their medical schools. On the first dimension- guidance in the field of medicine: the highest proportion (59.2%) of the medical students were strongly agreed on the positive role of medical instruction in their academic career and professional development. Similarly, the highest proportion were agreed (33.9% strongly agreed and 27.1% agreed) on the role of medical instruction to get broader insight in different medical specialties. There was also positive opinion on the role of guidance to increase awareness on professional responsibilities (36.3% strongly agreed and 30.7% agreed).

**Table 2:** Medical students’ attitudes towards medical instruction

Statements on mentoring in field of medicine	NO(%)	St. Disagree No(%)	Disagree No(%)	Neutral No(%)	Agree No(%)	St. Agree No(%)
I. Guidance in field of medicine						
1. Medical instruction plays an important role on medical students’ career choice and professional development	903(94.2)	17(1.8)	14(1.5)	54(5.6)	250(26.1)	568(59.2)
2. Medical instruction supports to reduce stress experience to practice medicine	768(80.1)	18(1.9)	38(4.0)	92(9.6)	267(27.8)	353(36.8)
3. Medical instruction supports medical students to get broader insight on various specialty areas	764(79.7)	24(2.5)	35(3.6)	120(12.5)	260(27.1)	325(33.9)
4. Medical instruction increases medical students’ awareness on professional responsibilities	711(80.4)	20(2.1)	31(3.2)	78(8.1)	294(30.7)	348(36.3)
Composite score	730(76.1)					
II. Professional development						
1. Mentoring stimulates medical students’ interest towards a certain clinical specialty area	908(94.7)	12(1.3)	22(2.3)	105(10.9)	376(39.2)	393(41.0)
2. Instructors serve as role model to obtain the required professional skills	912(95.1)	18(1.9)	23(2.4)	116(12.1)	378(39.4)	377(39.3)
3. Instructors serve as a role model to specialize in a particular clinical specialty area	900(93.8)	18(1.9)	26(2.7)	125(13.0)	391(40.8)	340(35.5)
Composite score	882(92)					
III. Orientation on research undertaking and ethics						
1. Here the medical school gives special attention on ethical issues	902(94.1)	47(4.9)	128(13.3)	263(27.4)	289(30.1)	175(18.2)
2. Medical instruction stimulate students’ interest towards research oriented careers	880(91.8)	37(3.9)	85(8.9)	249(26.0)	310(32.3)	199(20.8)
3. Teaching–learning process encourages creative thinking and taking an active role in new discovery	911(95.0)	37(3.9)	80(8.3)	219(22.8)	354(36.9)	221(23.0)
Composite score	853(88.9)					
The teaching- learning process						
1. Instructors commit their time and energy on a regular bases in teaching medical students	899(93.7)	67(7.0)	188(19.6)	249(26.0)	291(30.3)	104(10.8)
2. Provide feedback in constructive and caring manner	894(93.2)	83(8.7)	159(16.6)	220(22.9)	323(33.7)	109(11.4)
3. Non-judgmental and accepts individual differences	897(93.5)	92(9.6)	167(17.4)	246(25.7)	276(28.8)	116(12.1)
4. Mentoring help to enhance clinical skills and professionalism	882 (92.0)	51(5.3)	95(9.9)	203(21.2)	343(35.8)	190(19.8)
5. Instructors assist medical students in developing professional identity	886(92.4)	59(6.2)	126(13.1)	250(26.1)	319(33.3)	132(13.8)
Composite score	823(85.8)					
IV. Orientation towards in country practice						
1. The process encourages the students’ interest in pursuing their career within country	900(93.8)	47(4.9)	100(10.4)	261(27.2)	322(33.6)	170(17.7)
2. The training encourage to pursuing a career in an areas that country has shortages of qualified physicians	896(93.4)	39(4.1)	99(10.3)	277(28.9)	335(34.9)	146(15.2)
3. Medical instruction encourages medical students to work in the rural/distant part of the country	882(92.0)	50(5.2)	144(15.0)	327(34.1)	222(23.1)	139(14.5)
Composite score	871(90.8)					

With regards to professional development: the highest proportion were agreed (41.0% strongly agreed and 39.2% agreed) as their instructors could influence their interest towards a certain clinical specialty. Besides, a reasonable proportion of medical students strongly agreed/agreed on the fact that they made their instructors role models to acquire professional skills (78.9%) and to specialize in a particular medical specialty (76.3%).

Orientation in relation to research undertaking and ethics: nearly half of the medical students (18.2% strongly agree and 30.1% agree) agreed on the role of medical schools in giving special attention to ethical issues. However, nearly one third of the medical students were not in favor of the statement *‘the medical school encourage creative thinking and new discoveries’.*

With regards to the teaching-learning process: the highest proportion agreed (30.3%) on their instructors’ commitment to teach medical students, however nearly one fourth of them had neutral (26%) opinion about it. Similar level of agreement (33.7% agreed and 22.9% neutral) was observed on the way how their instructors provide feedbacks.

Moreover, concerning the role of medical schools towards within the country practice: the medical education has been playing limited role to influence students’ interest to pursue their postgraduate training within the country, which was agreed only by (33.6%) and quite a lot (27.2%) had neutral opinion about it. Similarly, on inspiring them towards an area that the country has shortages of specialists were agreed by (34.9%) but considerable number of them were neutral (28.9%). On rural practice, highest proportion had neutral (34.1%) stand towards the role of their medical schools in preparing the students to work in the rural/remote places of Ethiopia.

Table 3, shows findings from univariate and binary logistic regression analysis. In the univariate analysis gender, place of birth, parent’s educational level (father’s), medical school and the desire to serve within the country were statistically significantly (*p* < 0.05) associated with medical students’ preference to work in rural and remote areas. However, when binary logistic regression model was fitted to identify the potential predictor variables after controlling other variables, no statistical significant difference was observed in work place preference by parents’ education (*p* > 0.05). However, a significantly increased odds (AOR:1.55; 95%CI; 1.05,2.28) of intention to work in rural and remote places was found among male medical students than females. Similarly, those who were born in rural places have a significantly increased odds (AOR:1.52; 95%CI; 1.03, 2.25) of intention to work in rural and remote areas than those who were born in urban areas. Furthermore, there was significantly increased odds of intention to work in rural and remote places among the medical students of Addis Ababa University (AOR: 2.34, 95%CI; 1.64, 3.34) than the students in the other medical schools. And also the odds of intention to work in rural and remote places among those who had the desire to serve within the country was higher than their counterparts (AOR: 1.62, 95%CI; 1.18, 2.25).

**Table 3:** Results of univariate and binary logistic regression for predictors of medical student’s intention to work in rural and remote areas of Ethiopia (*N* = 784)

Predictor variables		Intended place		OR (95% CI)	AOR (95% CI)
		Remote/Rural *n* = 397	Urban *n* = 387		
Gender	Female	89	129	1	1
	Male	308	258	1.73(1.26, 2.38)*	1.55(1.05, 2.28)*
Age in years	>25	177	168	1	1
	<=25	219	219	0.95(0.71, 1.26)	0.96(0.68, 1.35)
Place of birth	Urban	215	262	1	1
	Rural	162	106	1.86(1.37, 2.52)**	1.52(1.03, 2.25)*
Parent education (of father)	> = 9th grade	219	250	1	1
	<=8th grade	160	117	1.56(1.16, 2.10)*	1.14 (0.77, 1.69)
Year of study	Sixth (Final)	145	166	1	1
	Fifth (C-II)	251	221	1.3(0.98, 1.73)	1.10 (0.77, 1.55)
Medical school	Others	243	283	1	1
	AAU	152	100	1.77(1.31, 2.40)**	2.34(1.64, 3.34)**
Desire to serve within the country	No	161	198	1	1
	Yes	221	172	1.58(1.19, 2.11)**	1.62(1.18, 2.25)*
Intention to leave	High	249	263	1	1
	Low	119	106	0.84(0.62, 1.15)	0.91(0.64,1.30)

**p < 0.001, *p < 0.05; OR: Odds ratio, AOR: Adjusted odds ratio; CI: confidence interval, AAU: Addis Ababa university

## Discussion

This study generated evidences on medical students’ career choices, role of medical education and their intention to work in rural and remote areas by involving senior medical students from six medical schools in Ethiopia as study participants. The majority of the medical students had the intention to practice in clinical/patient care settings. However, most want to practice in tertiary/teaching hospitals and the private sector than in general and primary hospitals, and also want to work in big cities. Internal medicine was the first cited career choice to specialize by most students followed by surgery. Survey items used to examine the role of medical instruction which relate to physician workforce preparation had strong internal consistency.

The fact that the majority want to practice in large hospitals and urban centers might suggest preferring the place where alternative opportunities and better working environment is available over those these things are not available [25]. The other possible explanation might be the existing medical education curriculum has limited role in preparing the students to serve in the rural places [24, 26] which rather leads to the choice of working in affluent communities [20, 27]. In addition, this might imply that in spite of good supply in physician workforce, geographic variations and imbalanced distributions might remain a challenge in Ethiopia, since intrinsic motivation plays an important role in health workers’ decision to work in the rural areas [28].

In human resources for health (HRH), skills mix is very essential. In the study, considerable size of the medical students was interested in very limited clinical areas to specialize (internal medicine, and surgery). Similarly, the need for proper career guidance for medical students in line with the national HRH needs was reported from Nigeria [29]. Their main reasons have also similarity with previous studies, which indicate the role of intellectual content, individual’s competencies, anticipated income, job value, and influence from others on specialty choices [30, 31]. In addition, this might be dependent on medical education workforce composition, as most students were in favor of the statements on professional development (*stimulating medical students’ interest towards a certain clinical specialty, instructors serve as role models to obtain professional skills and to specialize in a particular clinical specialty).* This might imply attentions need to be given for medical education workforce composition, faculty value and institutional culture in the medical schools [8, 32].

The medical schools play important roles to influence students’ attitudes in addition to other interrelated factors [6, 7]. However, in this study, in spite of strong internal consistency (the items used to examine the role of instruction), the medical instruction was not influencing medical students’ attitudes towards working in rural places. This might suggest the presence of limited attention in preparing them to work in rural and remote areas, which imply the needed attention in medical workforce preparation along with the rapid expansion in medical education, since most Ethiopians are rural dwellers.

Moreover, in the regression model: males, those born in rural areas, and those from AAU have an increased odds of preference to work in rural and remote areas. Similar findings are available in previous studies where gender, residence during high school, and parents’ residence were found to be significantly associated with attitudes towards working in rural areas [25]. These findings might also indicate the need for strategic approaches in strengthening rural-based medical training to inspire medical students to work in rural and remote areas [33]. Intensifying the desire to serve within the country can also potentially decrease the odds of working in urban areas.

### Limitation

One limitation of this study is behavioral intention might not be converted into practice by its very nature. In addition, this study did not include medical students from the private sector.

## Conclusion

In conclusion, majority of the medical students intend to practice in clinical/patient care settings. Nevertheless, a higher proportion of them intended to work in urban than the rural and remote locations, this might be an area of concern in medical workforce preparation and future direction. In addition, medical students’ inclination towards very few clinical specialty areas might suggest the role of medical instruction and/or shortages of instructors in various clinical specialty areas.

Furthermore, the internal consistency of new survey items used to examine medical instruction might indicate the need for using context based evidences in dealing with health workforce preparation in addition to illustrating the existing limited medical schools’ role in preparing students to work in rural and remote places. Therefore, the medical schools and the government of Ethiopia should give due consideration not only to equip the students with clinical skills but also to influence their attitudes to work in rural and remote locations and specialize in diverse clinical specialties.

## Additional file

Additional file 1: Questionnaires. (DOCX 40 kb)
