# Supplementary material for: Medical students’ career choices, preference for placement, and attitudes towards the role of medical instruction in Ethiopia
Source: BMC Med Educ. 2017 May 30;17:96. doi: 10.1186/s12909-017-0934-z (PMC5450253; doi:10.1186/s12909-017-0934-z)
Supplement: Additional file 1: — Questionnaires. (DOCX 40 kb) [file 12909_2017_934_MOESM1_ESM.docx]

## Questionnaires for Medical Students Career Plan in Ethiopia

Name of the university/college: ______________________________

**General Instruction: Please indicate your response on the space provided corresponding to the options.**

| **1.. Socio-Demographic Information** | | | | | | | | | | | | |
| --- | --- | --- | --- | --- | --- | --- | --- | --- | --- | --- | --- | --- |
| **No** | | **Questions** | **Response level** | | | | | | | | | |
|  | | Gender | - Male | | | | - Female | | | | | |
|  | | Date of birth | …. day…. Month …. .year | | | | | | | | | |
|  | | Place of Birth | - Regional town/city - Zonal town - Woreda town - Rural kebele | | | | | | | | | |
|  | | The region you came from? | - Addis Ababa - Amhara - Oromia - Dir Dawa | | | | | - SNNPR - Tigray - Other regions (Afar, Harari, Gambela, ) | | | | |
|  | | Please indicate your *year of study* | - C-2 - Medical intern | | | | | | | | | |
| **2. Household Characteristics** | | | | | | | | | | | | |
|  | **What is your parents’ highest level of education?** | | | | | | | | | | | |
|  | **Mother’s**   - - No education   - Primary school(grade 1-4)   - Junior school (grade 5-8)   - Secondary school (9-12)   - Diploma   - First degree and above | | | | | **Father’s**   - - No education   - Primary school(grade 1-4)   - Junior school (grade 5-8)   - Secondary school (9-12)   - Diploma   - First degree and above | | | | | | |
|  | What are your parents’ occupations for most of their lives? | | | | | | | | | | | |
|  | **Mother’s occupation** | | | **Father’s occupation** | | | | | | | | |
|  | - - Government employ   - Private/NGO employ   - Housewife   - Farmer   - Self-employ   - Other (specify)………………….. | | | - - Government employ   - Private/NGO employ   - Farmer   - Self-employ   - Other Specify…………………… | | | | | | | | |
| **3. Medical Students Career Plan** | | | | | | | | | | | | |
|  | | When you graduate from medical school, which one of the alternatives is the main focus of your career? | | | - - Patient/clinical care   - University /Scientific research   - Management position   - Other (specify)…………………… | | | | | | | |
|  | | Following is a list of work settings. Check the category which best describes the setting where you are interested to work with? | | | - Private clinic/hospital - NGOs- non clinical/ management - Teaching or university hospital - Zonal hospital - District hospital - Administrative office (government) - Other (please specify)………… | | | | | | | |
|  | | The first place that you want to initially practice medicine in Ethiopia?  ( Just after you graduate) | | | - Large cities (Addis Ababa, Hawassa, Bahrdar) - Zonal towns - Small towns (woreda towns) - Remote communities underserved (Gambela, Afar.) - Anywhere - I do not want to practice within the country at all - Other (Specify)……………………. | | | | | | | |
|  | | Following is a list of career. Which one are your first three career choices in which you are interested to specialize?  *(mark the first three)* | | | - Internal Medicine - Surgery - Pediatrics - Radiology - Gyn and Obs - Public health - Orthopedics | | | | | - Ophthalmology - Dermatology - Psychiatry - ENT - Anesthesiology - Family medicine - Other (Specify)…….. | | |
|  | | What are the three most important reasons that lead you to select the above field of specialization?*(Please specify only three alternatives in order of importance, mark the most important reason 1, the second most important 2, the third most important 3)* | | | - Availability of positions ..…… - Possibility of working abroad …. - Professional prestige ……… - Personal interest ………. - Income potential ……….. - Length of study………. - Influence from mentors …… - Other (please specify)……… | | | | | | | |
|  | | For the above career plan you have chosen in Q4. Where do you want to pursue/ receive the training? | | | - Within the country - Outside the country | | | | | | If your Ans**. Is within the country pls**. skip to **Q next section** |  |
|  | | If your first choice is outside the country, which country is your first choice? (Multiple response is possible) | | | - USA - Canada - UK | | | | - Australia - Other Europe countries - Other African countries - Other (specify)………… | | | |

**4. Section on medical education**

**Brief description: Instruction in field of medicine:** is a process whereby an experienced (the mentor/instructor) guides medical students (the mentee) in the development of their own ideas in **clinical knowledge, skills and personal** and professional development through formal and informal approaches **(**lab. sessions, during inpatient clerk, bedside teaching, morning sessions, round, research activity and so on) to equip them with required professional skills and guidance in their career choices.

**Instruction: Please put (√) mark corresponding to the option you agreed. While you choose the response please consider the situation which reflects in your medical school best.**

| **Question Statements** | **Strongly agree** | **Agree** | **Neutral** | **Disagree** | **Strongly Disagree** |
| --- | --- | --- | --- | --- | --- |
| **Guidance in field of medicine** |  |  |  |  |  |
| 1. Medical instruction plays an important role on medical students’ career choice and professional development |  |  |  |  |  |
| 1. Medical instruction supports to reduce stress experience to practice medicine |  |  |  |  |  |
| 1. Medical instruction supports medical students to get broader insight on various specialty areas |  |  |  |  |  |
| 1. Medical instruction increases medical students’ awareness on professional responsibilities |  |  |  |  |  |
| **Professional development** |  |  |  |  |  |
| 1. Mentoring stimulates medical students’ interest towards a certain clinical specialty area |  |  |  |  |  |
| 1. Instructors serve as role model to obtain the required professional skills |  |  |  |  |  |
| 1. Instructors serve as a role model to specialize in a particular clinical specialty area |  |  |  |  |  |
| **Orientation on research undertaking and ethics** |  |  |  |  |  |
| 1. Here the medical school gives special attention on ethical issues |  |  |  |  |  |
| 1. Medical instruction stimulate students’ interest towards research oriented careers |  |  |  |  |  |
| 1. Teaching–learning process encourages creative thinking and taking an active role in new discovery |  |  |  |  |  |
| **The teaching- learning process** |  |  |  |  |  |
| 1. Instructors commit their time and energy on a regular bases in teaching medical students |  |  |  |  |  |
| 1. Provide feedback in constructive and caring manner |  |  |  |  |  |
| 1. Non-judgmental and accepts individual differences |  |  |  |  |  |
| 1. Mentoring help to enhance clinical skills and professionalism |  |  |  |  |  |
| 1. Instructors assist medical students in developing professional identity |  |  |  |  |  |
| **Orientation towards in country practice** |  |  |  |  |  |
| 1. The process encourages the students' interest in pursuing their career within country |  |  |  |  |  |
| 1. The training encourage to pursuing a career in an areas that country has shortages of qualified physicians |  |  |  |  |  |
| 1. Medical instruction encourages medical students to work in the rural/distant part of the country |  |  |  |  |  |

**Thank You for Your Time and Participation!!!**
